# A Comprehensive Functional Investigation of the Human Translocator Protein 18 kDa (TSPO) in a Novel Human Neuronal Cell Knockout Model

**DOI:** 10.3390/ijms252312882

**Published:** 2024-11-29

**Authors:** Stefanie Bader, Tatjana Jahner, Anett Dörfelt, Doris Melchner, Iseline Cardon, Heiko I. Siegmund, Christoph Brochhausen, Rainer Rupprecht, Vladimir M. Milenkovic, Christian H. Wetzel

**Affiliations:** 1 Department of Psychiatry and Psychotherapy, University of Regensburg, 93053 Regensburg, Germany; stefanie.bader@ukr.de (S.B.); vladimir.milenkovic@ukr.de (V.M.M.); 2 Institute of Pathology, University of Regensburg, 93053 Regensburg, Germany; 3 Institute of Pathology, University Medical Center Mannheim, 68167 Mannheim, Germany

**Keywords:** mitochondrial function, TSPO, neurosteroids, induced pluripotent stem cells, CRISPR/Cas9, bioenergetics, ROS, calcium signalling, major depression

## Abstract

The translocator protein 18 kDa (TSPO) is a multifunctional outer mitochondrial membrane protein associated with various aspects of mitochondrial physiology and multiple roles in health and disease. Here, we aimed to analyse the role of TSPO in the regulation of mitochondrial and cellular functions in a human neuronal cell model. We used the CRISPR/Cas9 technology and generated TSPO knockout (KO) and control (CTRL) variants of human-induced pluripotent stem cells (hiPSCs). In a multimodal phenotyping approach, we investigated cellular and mitochondrial functions in neural progenitor cells (NPCs), astrocytes, and neurons differentiated from hiPSC CTRL and TSPO KO cell lines. Our analysis revealed reduced mitochondrial respiration and glycolysis, altered Ca^2+^ levels in the cytosol and mitochondrial matrix, a depolarised MMP, and increased levels of reactive oxygen species, as well as a reduced cell size. Notably, TSPO deficiency was accompanied by reduced expression of the voltage-dependent anion channel (VDAC). We also observed a reduced TSPO and VDAC expression in cells derived from patients suffering from major depressive disorder (MDD). Considering the modulatory function of TSPO and the similar functional phenotype of cells derived from patients with depression, we discuss a role of TSPO in the etiology or pathology of MDD. In summary, our findings indicate a general impairment of mitochondrial function in TSPO knockout (KO) cells. This deepens our insight into the intricate role of TSPO in a range of physiological and pathological processes.

## 1. Introduction

The translocator protein 18 kDa (TSPO) is a highly conserved multifunctional protein residing in the outer membrane of mitochondria (outer mitochondrial membrane, OMM) [1]. Originally described as peripheral-type benzodiazepine receptor (PBR), it was renamed translocator protein to reflect its suspected role in the transport of cholesterol into the mitochondria in the de novo generation of steroids. In addition to its role in cholesterol transport and steroid synthesis [2,3,4,5], TSPO has been shown to be involved in the regulation of various cellular and mitochondrial functions, such as mitochondrial bioenergetics (the oxidative phosphorylation system, OXPHOS, and the provision of ATP) [6,7,8], the beta-oxidation of fatty acids [9], the production of ROS [10], and Ca^2+^ homeostasis [7,8,11]. Moreover, TSPO is involved in regulating cellular downstream processes such as proliferation, survival, and apoptosis [12,13,14] and plays multiple roles in health and disease, ranging from neurodegeneration and neoplasia to psychiatric disorders [15,16]. TSPO is expressed to varying degrees in multiple tissues, with the highest levels of expression found in steroidogenic tissues and relatively low expression in the central nervous system. In the brain, TSPO can be detected predominantly in microglia and reactive astrocytes [17], where its expression is upregulated in the context of inflammation, neurodegeneration, and malignant neoplasia [12,15]. Steroids (especially neuroactive steroids) are important modulators of stress and stress-related disorders. At the subcellular level, steroid synthesis originates in the mitochondria, which places these organelles and the TSPO-associated functions at the centre of stress-related disorders [18,19,20]. In the literature, differing roles and functions of TSPO and TSPO ligands in various species and tissues have been reported [21,22,23]. The diverse functions of TSPO are likely mediated by a TSPO-containing protein complex, whose composition of interacting protein partners could be adapted to meet specific needs in various tissues and physiological or pathophysiological conditions. Among the many candidates, VDAC, a versatile channel for all kinds of ions and metabolites, seems to be one of the most important interaction partners [24,25]. To investigate the effect of TSPO expression or deficiency in the human neural system, we used CRISPR/Cas9 to generate TSPO-deficient induced pluripotent stem cells (iPSCs) reprogrammed from dermal fibroblasts obtained from a healthy donor. Furthermore, we differentiated the TSPO-deficient or TSPO-expressing iPSCs into neural progenitors, neurons, and astrocytes and performed a comprehensive analysis of mitochondrial function in the respective lines. We assessed steroid synthesis, mitochondrial respiration (the oxidative phosphorylation system, OXPHOS), glycolysis, mitochondrial membrane potential, cytosolic and mitochondrial Ca^2+^ and reactive oxygen species (ROS) levels, expression of inflammation- and mitochondrial dynamic-associated genes, general mitochondrial content, and cell size. Our findings point to a central role of TSPO in the regulation of mitochondrial and cellular functions, underlining the significance of this protein in physiological and pathophysiological states. Confirming that alterations in mitochondrial functions are also evident in a human cell model of depression [26,27,28], we extended our studies to fibroblasts of patients with depression and found reduced expressions of both TSPO and VDAC in MDD fibroblasts, highlighting a possible causal link between TSPO and depression.

## 2. Results

### 2.1. Confirmation of TSPO Knockout in hiPSCs

To study the role and function of TSPO in neural cells, CRISPR/Cas9-mediated TSPO knockout variants were generated from hiPSCs reprogrammed from primary skin fibroblasts of a healthy donor. Knockouts were confirmed by DNA sequencing of PCR products and Western blot analysis.

**Figure 1:**
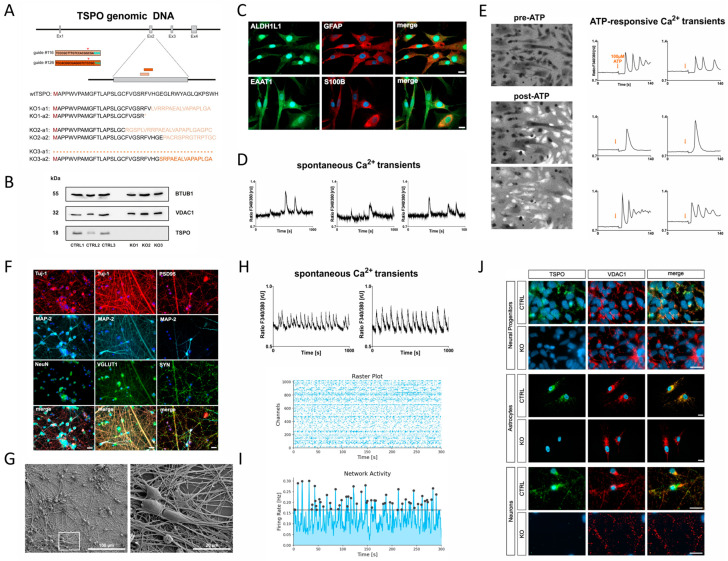
Characterisation of TSPO knockout model. (**A**) Translocator protein 18 kDa (TSPO) gene deletion in human-induced pluripotent stem cells. Schematic overview displaying the CRISPR/Cas9 sgRNA #116 and #126 targeting sites on exon 2 of the TSPO gene, along with their respective PAM sequences (cyan). Asterisk * represents end of translation (stop codon). Successful TSPO knockout in both alleles was confirmed using DNA sequencing. (**B**) Western blot analysis of TSPO protein expression in hiPSC control (CTRL1, CTRL2, and CTRL3) and knockout (KO1, KO2, and KO3) lines, demonstrating a complete loss of TSPO. BTUB1 was used as a loading control. (**C**) Immunofluorescence staining demonstrates the presence of mature astrocyte markers, including GFAP, ALDH1L1, S100β, and EAAT1. Scale bar represents 20 µm. Representative examples of (**D**) spontaneous and (**E**) ATP-responsive Ca^2+^ transients in astrocytes loaded with Fura-2/AM. In (**E**), the gliotransmitter ATP was added at a final concentration of 100 µM. Representative Fura-2 ratio images display brighter cells post-ATP treatment, indicating increased intracellular calcium levels (left). (**F**–**H**) Characterisation of hiPSC-derived neurons. (**F**) Immunofluorescence staining at day 21 of differentiation reveals the expression of neuronal markers in the induced neurons. The neurons display typical neuronal cytoskeleton proteins MAP-2 and Tuj-1, as well as the presynaptic synaptophysin (SYN) and post-mitotic neuronal nuclear marker NeuN. Immunostaining for VGLUT1 and postsynaptic PSD95 point to glutamatergic synapses. Scale bar indicates 20 µM. (**G**) Electron micrographs provide high-resolution visualisation of neuronal morphology after 21 days of differentiation. (**H**) Changes and oscillations in intracellular Ca^2+^ concentrations visualised via Fura-2/AM live-cell imaging demonstrate the spontaneous activity of induced neurons at day 21 of differentiation. (**I**) First signs of a synchronous activity pattern assessed with the HD-MEA system from Maxwell technologies at day 30 of differentiation. (**J**) TSPO knockout in hiPSC-derived neural progenitors, astrocytes, and neurons. TSPO gene deletion was further confirmed by TSPO antibody co-staining, using VDAC1 as a mitochondrial marker. Scale bar indicates 20 µm. Control of TSPO immunofluorescence (without 1st antibody) is shown in Supplementary Figure S2.

In one of the clones (KO1), the nucleofection of the cAB03 plasmid containing the sgRNA-targeting sequence #116 deleted a 17 bp fragment, which resulted in a shift in the open reading frame (ORF) after the amino acid V26 in allele 1. An additional 246 bp insertion in allele 2 generated a stop codon (*) after residue R24. A second clone (KO2) demonstrated a 26 bp deletion in allele 1 and a deletion of a 16 bp fragment in allele 2, resulting in a frameshift and generation of a non-functional *TSPO* gene. Electroporation of hiPSCs with the cAB03 plasmid, including the #126 targeting sequence, generated a third clone (KO3), which lost the complete exon 2 and adjacent bases (286 bp) in allele 1. The deletion of 19 bp in allele 2 led again to a shift in the ORF and a non-functional *TSPO* gene (Figure 1A).

Western blot analysis of TSPO protein expression in all three clones revealed a complete loss of TSPO in the mutated cell lines KO1, KO2, and KO3, whereas the corresponding isogenic control cell lines CTRL1, CTRL2, and CTRL3 exhibited prominent TSPO expression at 18 kDa (Figure 1B).

### 2.2. Differentiation of hiPSC-Derived NPCs into Astrocytes

iPSCs were differentiated into NPCs and stained for the neural progenitor markers SOX2 and PAX6 [29]. Supplementary Table S1 shows that most cells co-express both markers and could, therefore, be considered neural progenitors. Furthermore, astrocytes and induced cortical-like neurons were generated by differentiating NPCs following the protocol described by [30] and [31], respectively.

To identify and confirm the maturity of the astrocytes (Astro), the expression of specific markers was examined by immunostaining. Within 30 days, hiPSC-derived astrocytes were immunopositive for glial fibrillary acidic protein (GFAP), S100 calcium-binding protein B (S100β), glutamate transporter excitatory amino acid transporter 1 (EAAT-1), and aldehyde dehydrogenase 1 family member L1 (ALDH1L1) (Figure 1C).

The functionality of mature astrocytes was verified by the presence of spontaneous and ATP-elicited Ca^2+^ transients, as previously described for astrocytes [30,32,33,34,35]. The Ca^2+^ indicator Fura-2/AM was used to monitor Ca^2+^ signalling under basal conditions and in response to a pulse of extracellular ATP (100 µM) [33]. While some cells showed already spontaneous Ca^2+^ spikes (Figure 1D), a single pulse of the gliotransmitter ATP produced a slow Ca^2+^ response in most cells (Figure 1E).

### 2.3. Differentiation of hiPSC-Derived NPCs into Neurons

To confirm the identity of hiPSC-derived neurons, the expression of specific neuronal markers was investigated using immunocytochemical analysis. Somato-dendritic microtubule-associated protein 2 (MAP-2)- and class III β-tubulin (Tuj-1)-positive neuronal cells were detected after 21 days of differentiation, labelling the neurite network. Additionally, visualisation of the post-mitotic nuclei using the neuronal marker NeuN, co-localising with the nuclear-specific dye Hoechst, indicated a highly pure neuronal culture (Supplementary Figure S1). The induced neurons displayed mature synaptic terminals, as indicated by distinct punctate labelling with presynaptic (synaptophysin, VGLUT1) and postsynaptic (PSD95) markers (Figure 1F). The vesicular glutamate transporter (VGLUT) and the scaffolding postsynaptic density protein 95 (PSD95) serve as distinctive biomarkers for glutamatergic synapses, confirming that the cells are cortical-like glutamatergic neurons. To further visualise neuronal morphology, high-resolution electron micrographs were generated, revealing densely interconnected networks of neurites and protrusions (Figure 1G).

Excitability is a hallmark of neuronal function. Even cultured neurons tend to self-organize a functional network showing spontaneous activity [36]. As changes and oscillations in intracellular Ca^2+^ levels through voltage-dependent Ca^2+^ channels evoked by the electrical activity of individual neurons [37] are an important aspect of spontaneous activity, neuronal Ca^2+^ signalling was monitored using Fura-2/AM in hiPSC-derived neurons (Figure 1H). Additionally, growing hiPSC-derived neurons on high-density multielectrode arrays (HD-MEAs) and extracellular recording of electrical activity demonstrated synchronous and asynchronous patterns of neural network activity [32]. These preliminary experiments further confirmed successful differentiation into neurons (Figure 1I).

### 2.4. Confirmation of TSPO Knockout in NPCs, Astrocytes, and Neurons

TSPO knockout was confirmed in differentiated NPCs, astrocytes, and neurons using immunofluorescence. Anti-VDAC1 fluorescence clearly demonstrated the presence of a mitochondrial network in both the CTRL and KO cells. TSPO expression, as indicated by anti-TSPO antibody staining, was only visible in CTRL cells but was completely absent in KO cells, confirming the loss of TSPO expression in mutated NPCs, astrocytes, and neurons (Figure 1J).

### 2.5. Effect of TSPO Expression on Steroid Synthesis in hiPSC-Derived Astrocytes

**Figure 2:**
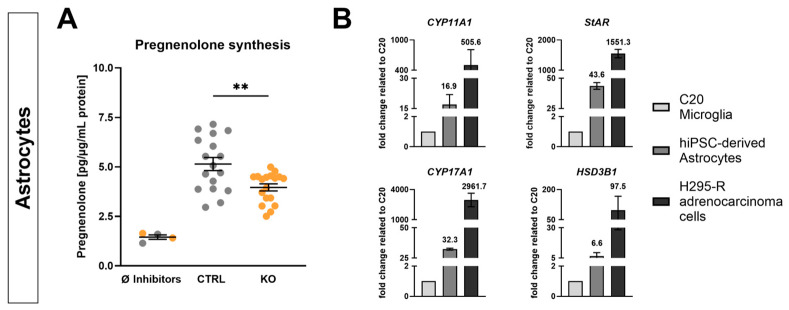
Pregnenolone synthesis and mRNA expression of steroidogenic enzymes. (**A**) TSPO knockout resulted in significantly lower basal pregnenolone production in hiPSC-derived astrocytes compared to their TSPO-expressing CTRL cells. Dot blots represent normalised concentrations ± SEM using independent samples *t*-test. (**B**) Relative mRNA levels of key steroidogenic proteins (CYP11A1, CYP17A1, HSD3B1, and StAR) in hiPSC-derived astrocytes (control) and the adrenocarcinoma cell line H295-R related to the very low gene expression in C20 microglial cells. Bar graphs represent the mean fold change ± SEM. Asterisks in the figure represent ** *p* < 0.01.

TSPO expression is elevated in glial cells and has been reported to be involved in mitochondrial cholesterol transport, thus implying a role in regulation of pregnenolone synthesis. To evaluate the involvement of TSPO in neurosteroidogenesis, the concentration of pregnenolone in hiPSC-derived astrocytes was investigated using ELISA. The supernatants collected from TSPO knockout cells contained significantly lower concentrations of pregnenolone than those from CTRL cells (3.961 ± 0.18 pg/µg/mL protein, *n* = 17 vs. 5.144 ± 0.33 pg/µg/mL protein, *n* = 18, *p* = 0.0031) after 3 h of incubation with the enzyme inhibitors trilostane (3β-hydroxysteroid dehydrogenase, HSD3B1) and SU10603 (17-OH-steroid hydroxylase, CYP17A1), which were used to prevent further metabolism of the acutely synthesised pregnenolone (Figure 2A). Omitting these inhibitors from the assay medium resulted in a low basal concentration of pregnenolone in both CTRL and TSPO KO cells. These data demonstrate that the hiPSC-derived astrocytes are capable of steroid production, which is regulated by the presence of TSPO.

To gain insight into the steroid-synthesising capacity of hiPSC-derived astrocytes, an analysis of gene expression was performed for key steroidogenic enzymes, including cytochrome P450 (*CYP11A1*), *HSD3B1*, and *CYP17A1*, which are crucial for the conversion of cholesterol to pregnenolone and subsequent neurosteroid production, as well as steroidogenic acute regulatory protein (*StAR*), which is essential for cholesterol transport. As shown in Figure 2B, the results demonstrate higher mRNA transcript levels of these proteins in control (i.e., TSPO-expressing) astrocytes than in the C20 microglial cell line, which does not produce substantial amounts of pregnenolone [7]. mRNA levels observed in hiPSC-derived astrocytes were still considerably lower than those found in the steroidogenic adrenocarcinoma cell line H295-R. Nevertheless, these findings support the potential importance of TSPO in the regulation of neurosteroidogenesis in astrocytes, as evidenced by the reduced pregnenolone concentration in the TSPO KO cells.

### 2.6. Impact of TSPO Expression on Mitochondrial Respiration

**Figure 3:**
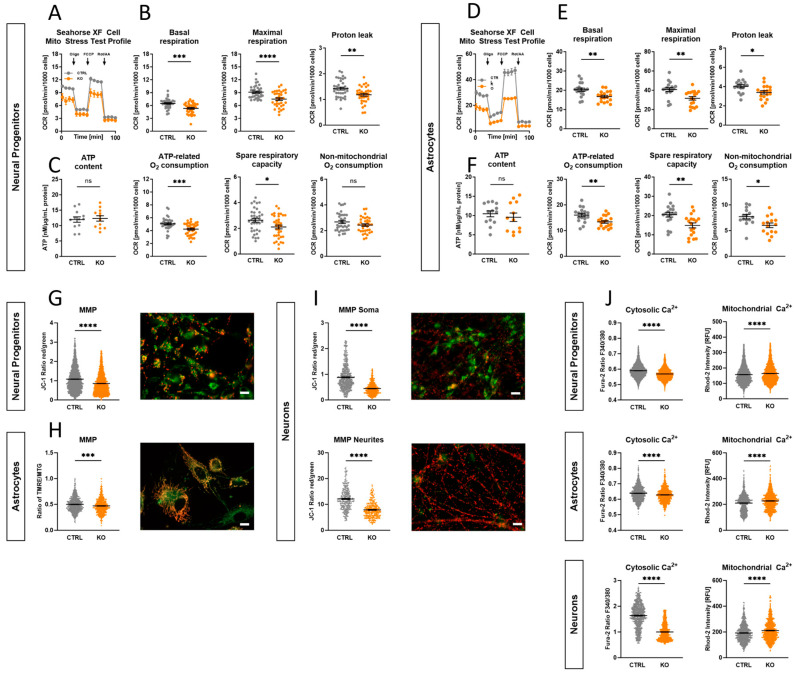
Mitochondrial respiration in TSPO knockout and control cells. The oxygen consumption rate (OCR) was measured in TSPO KO and CTRL cells to assess the basal conditions and the effects of sequential injections of oligomycin (Oligo), carbonyl cyanide-4-(trifluoromethoxy)-phenylhydrazone (FCCP), and rotenone/antimycin A (Rot/AA) targeting different complexes of the electron transport chain. Representative examples of kinetic profiles of the Agilent Seahorse XF Mito Stress Test are shown for (**A**) NPCs and (**D**) astrocytes. Key respiratory parameters, including basal OCR, maximal respiration, proton leak, ATP-related oxygen consumption, spare respiratory capacity, and non-mitochondrial respiration, were compared between the KO and CTRL groups in (**B**) NPCs and (**E**) astrocytes. Total ATP content was quantified in (**C**) NPCs and (**F**) astrocytes using a bioluminescence assay. Dot plots display the normalised mean OCR values for NPCs and astrocytes. ATP levels in nM normalised to the total protein amount [µg/mL] are shown for the ATP content in NPCs and astrocytes. Data are presented as mean ± SEM using independent samples *t*-test. (**G**–**I**) Effects of TSPO gene deletion on mitochondrial membrane potential. TSPO deficiency resulted in a significant reduction in mitochondrial membrane potential in knockout cells of hiPSC-derived neural progenitors (**G**), astrocytes (**H**), and neurons (**I**) (*p* < 0.0001, Mann–Whitney U test). The MMP in NPCs and neurons is indicated by the JC-1 fluorescence ratio of red/green (JC-1 aggregates/monomers) and in astrocytes by the ratio of TMRE/MTG fluorescence. Dot plots show mean red/green or TMRE/MTG ratio ± SEM of *n* = 3 biological replicates of several individual cell lines (NPC: CTRL *n* = 3, KO *n* = 3; Astro: CTRL *n* = 2, KO *n* = 2; Neuron: CTRL *n* = 2, KO *n* = 2). Representative fluorescence microscopy images of NPCs and neurons loaded with the cationic dye JC-1 and of astrocytes loaded with the cationic dye TMRE and the mitochondria-specific dye MitoTracker Green. Scale bar indicates 20 µm. (**J**) Impact of TSPO gene deletion on cytosolic and mitochondrial Ca^2+^. TSPO knockout cells showed significantly reduced cytosolic Ca^2+^ levels, as indicated by the Fura-2 fluorescence ratio F_340_/_380_, while the mitochondrial Ca^2+^ concentration was significantly increased in TSPO KO NPCs, astrocytes, and neurons, as indicated by Rhod-2 mean fluorescence intensity (MFI) ± SEM. Exact statistical values are given in Supplementary Table S4. Asterisks in the figure represent *p*-values as follows: * *p* ≤ 0.05, ** *p* < 0.01, *** *p* < 0.001, and **** *p* < 0.0001.

Mitochondria are crucial for the regulation of cellular bioenergetics by providing most of the cell’s energy through the oxidative phosphorylation system (OXPHOS). To investigate the functionality and performance of the OXPHOS, the oxygen consumption rate (OCR) was measured using extracellular flux analysis in CTRL and TSPO KO NPCs (Figure 3A,B) and astrocytes (Figure 3D,E), respectively. We found that TSPO expression significantly affected different aspects of mitochondrial respiration. KO cells of both cell types showed significantly lower basal respiration than TSPO-expressing CTRL cells (NPC: *p* = 0.0002; Astro: *p* = 0.0022). Moreover, maximal respiration in the presence of the uncoupler FCCP and the resulting spare respiratory capacity, which reflects the cell’s bioenergetic reserve and flexibility in meeting high energetic demands, were significantly reduced in TSPO-deficient cells (maximal respiration: NPC *p* < 0.0001; Astro *p* = 0.0019; spare respiratory capacity: NPC *p* = 0.0145; Astro *p* = 0.0039). Additionally, the oxygen consumption related to ATP production differed significantly between KO and CTRL neural progenitors and astrocytes (NPC *p* = 0.0004; Astro *p* = 0.003). The proton leak was also significantly lower in the knockout group than in the CTRLs (NPC *p* = 0.0031; Astro *p* = 0.0123).

To provide a more comprehensive understanding of OXPHOS activity and to study the bioenergetic core function of mitochondrial metabolism, the total ATP levels of the cells were measured. Interestingly, we did not detect a difference in cellular ATP content, neither in TSPO KO NPCs nor in astrocytes, compared with their respective controls (NPC *p* = 0.7834; Astro *p* = 0.5011) (Figure 3C,F).

The altered respiratory parameters in TSPO-deficient cells highlight the significance of TSPO in regulating mitochondrial bioenergetics in neural cells. However, the unaffected ATP levels suggest compensatory mechanisms in TSPO KO cells to maintain intracellular ATP levels, despite the potentially reduced energy production associated with decreased OXPHOS.

### 2.7. Role of TSPO in the Modulation of the Mitochondrial Membrane Potential

During OXPHOS, a proton gradient across the IMM contributes to the formation of the mitochondrial membrane potential (MMP), which provides valuable insights into mitochondrial function, metabolic activity, and potential imbalances arising from mitochondrial dysfunction.

To visualise the MMP in NPCs and neurons, mitochondria were labelled with the ratiometric cationic dye JC-1 (200 nM), which forms potential-dependent red fluorescent aggregates in highly energised mitochondria, whereas in response to the dissipation of the MMP, the dye molecules disaggregate into green fluorescing monomers. The MMP of hiPSC-derived astrocytes was assessed by loading the cells with a combination of the cationic dye TMRE (25 nM, non-quenching mode) and MitoTracker Green (MTG, 200 nM). TMRE also accumulates in negatively charged mitochondria to an extent that depends on the strength of the electric field. The mitochondria-specific dye MTG is nearly potential-independent and is used to normalise the TMRE fluorescence. The ratio of the fluorescence signals emitted by the two different states of JC-1 (red/green), as well as the ratio F_TMRE_/F_MTG_, is therefore a measure of the strength of the MMP [38].

TSPO deficiency in hiPSC-derived neural progenitors and astrocytes decreased the fluorescence ratio from 1.08 ± 0.01 and 0.49 ± 0.005 to 0.86 ± 0.01 and 0.47 ± 0.006, respectively (*p* < 0.0001) (Figure 3G,H). Given that the mitochondria residing in the soma or neurites of the neurons appeared at different focal planes, images of JC-1 fluorescence of the relevant structures were captured separately. The TSPO knockout neurons showed again a significantly reduced JC-1 ratio (*p* < 0.0001), especially in the neurites, thereby indicating a less hyperpolarised membrane potential in the mitochondria devoid of TSPO (soma 0.88 ± 0.23 vs. 0.45 ± 0.008; neurites 12.07 ± 0.23 vs. 7.85 ± 0.16) (Figure 3I). Overall, knocking out TSPO resulted in a robust and significant decline in the mitochondrial membrane potential across all examined cell types. This again indicates that the overall function of the electron transport chain (ETC); i.e., the translocation of protons, and the parallel backflow of protons through the ATP synthase, which determines the mitochondrial membrane potential, is diminished.

### 2.8. Involvement of TSPO in Ca^2+^ Homeostasis

Mitochondria play a critical role in Ca^2+^ homeostasis. Driven by their negative membrane potential, mitochondria attract and accumulate calcium ions within the matrix, thereby directly influencing and regulating cellular Ca^2+^ dynamics. Owing to their capability to store or release calcium ions, mitochondria serve as calcium buffers with both beneficial and detrimental effects. The MMP, along with Ca^2+^-conducting channels (e.g., voltage-dependent anion channel, VDAC) and the activity of Ca^2+^-transporting proteins in the IMM (e.g., mitochondrial calcium uniporter, MCU), influences the extent of mitochondrial Ca^2+^ uptake.

Using Ca^2+^-sensitive dyes, the cytosolic and mitochondrial Ca^2+^ levels were investigated. Cytosolic Ca^2+^ concentrations were measured by loading the cells with the ratiometric dye Fura-2/AM, whereas Rhod-2/AM was used to assess mitochondrial Ca^2+^ levels. Consistent within all analysed cell types, a significantly reduced Fura-2 ratio (F_340nm_/F_380nm_) was observed in the TSPO KO cell lines compared to the respective CTRL cells, showing decreased basal cytosolic Ca^2+^ levels (*p* < 0.0001) (Figure 3J). The Rhod-2 fluorescence intensity showed a significant increase amongst all KO cell lines (*p* < 0.0001), which is indicative of altered Ca^2+^ flux or active transport processes (Figure 3J).

TSPO is a highly interactive protein. It may function as a molecular hub of VDAC-containing protein complexes, thereby contributing to the maintenance of intracellular Ca^2+^ homeostasis.

**Figure 4:**
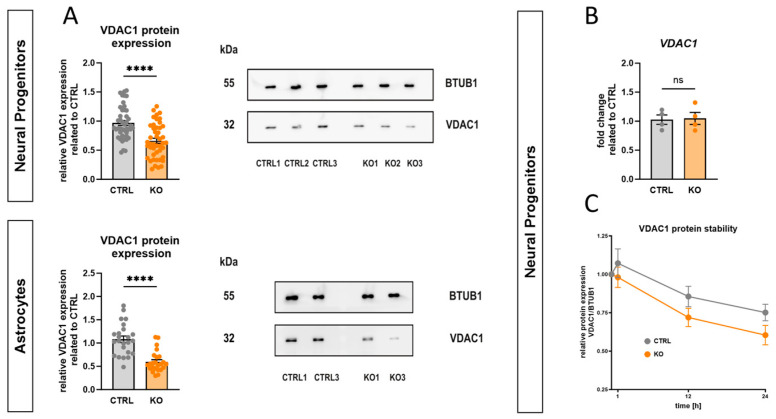
Effect of TSPO deficiency on VDAC1 gene expression and protein stability. (**A**) Deletion of *TSPO* gene led to decreased expression of VDAC1 protein in NPCs and astrocytes. Representative Western blots depict the expression of the housekeeper gene BTUB1 at 55 kDa and VDAC1 at 32 kDa in NPCs and astrocytes. Bar graphs show densitometric, relative mean of VDAC expression. Individual values were normalised to the respective expression level of BTUB1 and related to CTRL mean. Data are presented as mean ± SEM using Mann–Whitney U test. (**B**) qRT-PCR analysis revealed no significant differences in mRNA transcript levels of VDAC1 in TSPO knockout NPCs compared to CTRL cells. Bar graphs show fold change ± SEM related to CTRL of *n* = 2 biological replicates. (**C**) CTRL and KO cells were treated with cycloheximide (100 µg/mL) for the indicated times, and endogenous VDAC1 was detected using a specific anti-VDAC1 antibody. Values were normalised to the expression level of BTUB1 and were related to the initial VDAC1 protein level (0 h of CHX) for each group. Data are shown as relative mean ± SEM of *n* = 9 biological replicates. Asterisks in the figure represent **** *p* < 0.0001.

Western blot analyses with an anti-VDAC1 antibody showed that TSPO deficiency in hiPSC-derived NPCs and astrocytes was accompanied by a 33.5 ± 4.4% and 44.8 ± 4.2% reduction in VDAC1 protein expression, respectively, compared to the respective control (*p* < 0.0001, Mann–Whitney U test), as shown in Figure 4A. This finding supports the idea of functional and structural interactions between these two proteins. This altered VDAC1 expression suggests that changes in TSPO expression have direct or indirect effects on key physiological aspects of mitochondrial function.

To gain insight into the underlying mechanisms, an analysis of VDAC1 mRNA expression and protein stability was performed. qRT-PCR analysis indicated that loss of TSPO expression had no effect on VDAC1 transcription (Figure 4B).

To assess the stability of the endogenous VDAC1 protein, a cycloheximide (CHX) chase protein degradation assay was conducted. Therefore, neural progenitors were incubated with 100 µg/mL CHX, a well-known inhibitor of eukaryotic translation, for a maximum of 24 h to evaluate VDAC1 protein levels at different time points after CHX treatment. VDAC1 protein expression was quantified as a percentage of the initial VDAC1 protein level (0 h of CHX treatment) and was normalised to BTUB1 expression. Our findings indicate that the amount of VDAC1 protein in CTRL NPCs declined to 67.4 ± 3.8% of the initial value after 24 h, while the KO cells showed higher protein degradation (55.9 ± 5.6% of initial value), as shown in Figure 4C.

### 2.9. Impact of TSPO on Cellular Bioenergetics and Glycolysis

**Figure 5:**
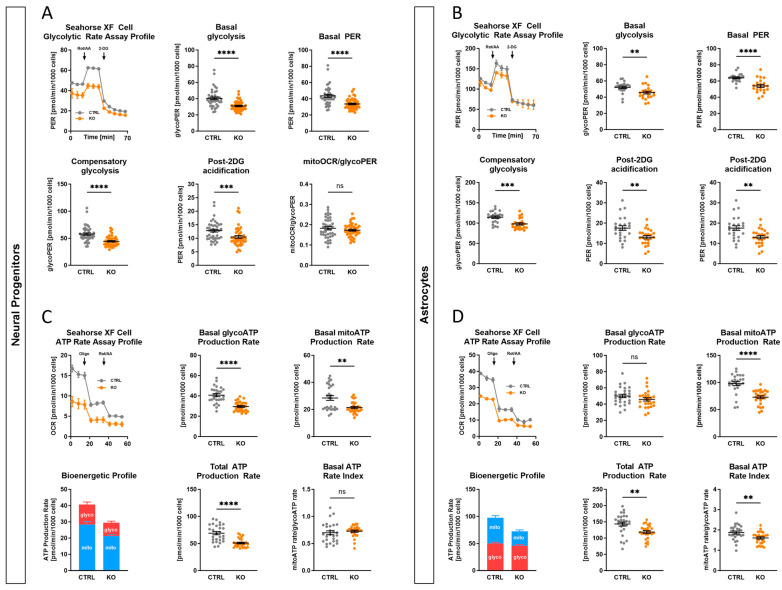
Glycolytic rates in TSPO knockout and control cells. Oxygen consumption rate (OCR) and extracellular acidification rate (ECAR) were measured in TSPO KO and CTRL cells. Following basal measurement of OCR and ECAR to determine the basal proton efflux rate (PER), rotenone and antimycin A (Rot/AA) were added to determine compensatory glycolysis and basal glycolysis (glycoPER) by blocking mitochondrial respiration. Subsequently, 2-deoxy-D-glucose (2-DG), an inhibitor of glycolysis by competitively binding hexokinase, was injected to ensure that the observed PER was caused by glycolysis. Representative kinetic profiles of the Agilent Seahorse Glycolytic Rate assay and key glycolytic parameters are shown for (**A**) neural progenitors (NPCs) and (**B**) astrocytes. Key glycolytic parameters, including basal glycolysis, basal PER, compensatory glycolysis, post-2DG acidification, and mitoOCR/glycoPER, were compared between KO and CTRL groups. Dot plots display normalised mean PER values ± SEM using independent samples *t*-test or Mann–Whitney U test for NPCs and astrocytes. (**C**,**D**) ATP production rates in TSPO knockout and control cells. Oxygen consumption rate (OCR) and extracellular acidification rate (ECAR) were measured, and ATP production rates were calculated for TSPO KO and CTRL cells after sequential addition of the metabolic modulators oligomycin (oligo) and rotenone/antimycin A (Rot/AA). Representative kinetic profiles and ATP production rates conducted with the Agilent Seahorse XFp Real-time ATP Rate assay are shown for (**C**) NPCs and (**D**) astrocytes. Dot plots display normalised mean ATP production rate ± SEM for NPCs and astrocytes using independent samples *t*-test or Mann–Whitney U test. Asterisks in the figure represent *p*-values as follows: ** *p* < 0.01, *** *p* < 0.001, and **** *p* < 0.0001.

In addition to OXPHOS, ATP production via the glycolytic pathway is an alternative way for the provision of energy. To measure the glycolytic flux, the proton efflux rates (PER) of the TSPO KO and CTRL groups were analysed. Using the Agilent Seahorse Glycolytic Rate Assay, TSPO KO neural progenitors (Figure 5A) and astrocytes (Figure 5B) showed significantly lower basal glycolysis (glycoPER) than CTRL cells (NPC *p* < 0.0001; Astro *p* = 0.0023). According to basal glycolysis, TSPO-deficient cells also had significantly reduced compensatory glycolysis (NPC *p* < 0.0001; Astro *p* = 0.0002). In addition, the basal PER and post-2-DG acidification rate of KO cells were also statistically significant different from those of CTRL cells (basal PER: NPC *p* < 0.0001; Astro *p* < 0.001; post-2-DG acidification: NPC *p* = 0.0005; Astro *p* = 0.00238). Conversely, the CTRL and KO groups did not differ significantly in their basal mitoOCR/glycoPER ratios (NPC *p* = 0.31; Astro *p* = 0.297).

Collectively, TSPO KO cells demonstrated lower basal respiration, spare respiratory capacity, glycolysis, and compensatory glycolysis in comparison with CTRL cells, but a similar mitoOCR/glycoPER ratio, indicating a lower metabolic flux in TSPO KO cells.

The ATP production rate is an effective means of characterising cellular metabolism. Using the Agilent Seahorse XFp Real-Time ATP Rate Assay, the total ATP production rates as well as the fractions of ATP produced through mitochondrial OXPHOS and glycolysis of hiPSC-derived neural progenitors and astrocytes were analysed. As shown in Figure 5C,D and consistent with the Seahorse Mito Stress Test, the mitochondrial-derived ATP production rate (basal mitoATP production rate) was significantly decreased in the TSPO KO group of both cell types (NPC *p* = 0.0035; Astro *p* < 0.0001). Regarding the glycolysis-specific ATP rate fraction, only TSPO deficiency in NPCs resulted in a significant reduction compared to that in the control cells (NPC *p* < 0.0001). The contribution of glycolysis to the total ATP production rate was not altered in KO and CTRL astrocytes (Astro *p* = 0.17).

However, consistent with the unchanged basal mitoOCR/glycoPER ratio, the overall metabolic flux, represented by the total ATP production rate, was lower in both TSPO-deficient NPCs and astrocytes than in TSPO-expressing controls (NPC *p* < 0.0001; Astro *p* = 0.002). The XF ATP Rate Index, a measure for detecting changes or differences in metabolic phenotypes, showed a significant decrease in TSPO KO astrocytes, indicating a less oxidative phenotype compared to CTRL cells (Astro *p* = 0.0051). As both mitochondrial and glycolytic ATP rates were decreased in TSPO-deficient NPCs, the XF ATP Rate Index remained unchanged (NPC *p* = 0.22). Taken together, these findings demonstrate the impact of TSPO expression on the bioenergetic capacity of cells and support the regulatory role of TSPO in cellular and mitochondrial energy homeostasis.

### 2.10. TSPO-Deficient Neural Progenitors and Astrocytes Show Oxidative Stress

**Figure 6:**
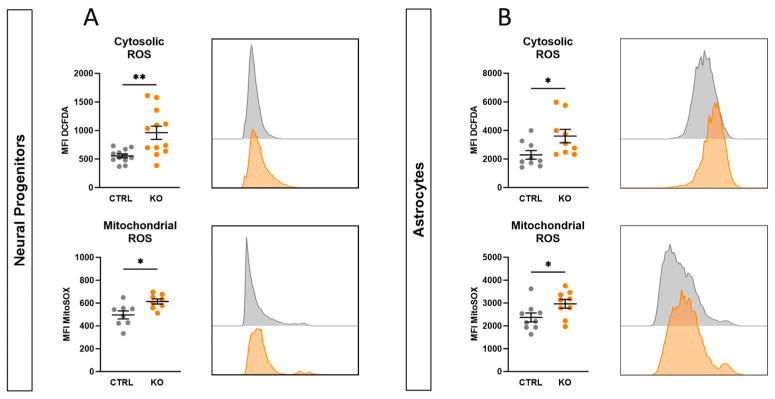
Oxidative stress indicators in TSPO knockout and control cells. Cytosolic reactive oxygen species (ROS) and mitochondrial ROS (superoxide) were measured in (**A**) NPCs and (**B**) astrocytes by flow cytometry and are indicated by DCFDA and MitoSOX mean fluorescence intensity (MFI). Dot blots show MFI ± SEM using independent samples *t*-test of *n* = 4 individual experiments; 1 × 10^5^ and 2 × 10^4^ events were recorded for each replicate of NPCs and astrocytes, respectively. Histograms indicate the frequencies of measured values in the fluorescence intensity bins. Asterisks in the figure represent *p*-values as follows: * *p* ≤ 0.05, ** *p* < 0.01.

Mitochondria exert a crucial role in maintaining the cell’s redox homeostasis. An imbalance between the production and detoxification of reactive oxygen species causes oxidative stress, leading to molecular and cellular damage. Cellular redox was analysed by flow cytometry using the fluorescent dyes DCFDA and MitoSOX^TM^ Red to detect global cytosolic hydrogen peroxide (H_2_O_2_), peroxyl radicals (HO_2_), and mitochondrial superoxide. As shown in Figure 6, both cytosolic and mitochondrial ROS were increased in TSPO knockout neural progenitors (cytosolic ROS *p* = 0.0028; mitochondrial ROS *p* = 0.013) (Figure 6A) and astrocytes (cytosolic ROS *p* = 0.031; mitochondrial ROS *p* = 0.046) (Figure 6B) compared with the respective controls, indicating oxidative stress and an imbalanced redox state of the cell.

### 2.11. Effect of TSPO-Deficiency on mtDNA Copy Number, Mitochondrial Content, and Cell Size

Mitochondria possess their own genetic material, circular mtDNA molecules, which encode some rRNA, tRNA, and essential components of the mitochondrial ETC. The dynamic equilibrium between mtDNA degradation and synthesis determines the mtDNA copy number, ranging from several hundreds to 10^4^ copies in different cell types. The quantity and quality of mtDNA directly affect mitochondrial function, with mtDNA copy number serving as an indicator of mitochondrial integrity. Levels of mtDNA copy number are correlated with energy reserves, oxidative stress, and changes in the MMP.

To evaluate whether the observed differences in the mitochondrial membrane potential and respiratory activity are related to lower mitochondrial content, the mtDNA copy number was determined in relation to the diploid nuclear genome in TSPO KO and CTRL cells. The relative mtDNA content was quantified using quantitative polymerase chain reaction (qRT-PCR) targeting the mitochondrial-encoded gene tRNA leucine 1 (mt-TL1) and the nuclear-encoded single-copy gene beta-2-microglobulin (B2M).

**Figure 7:**
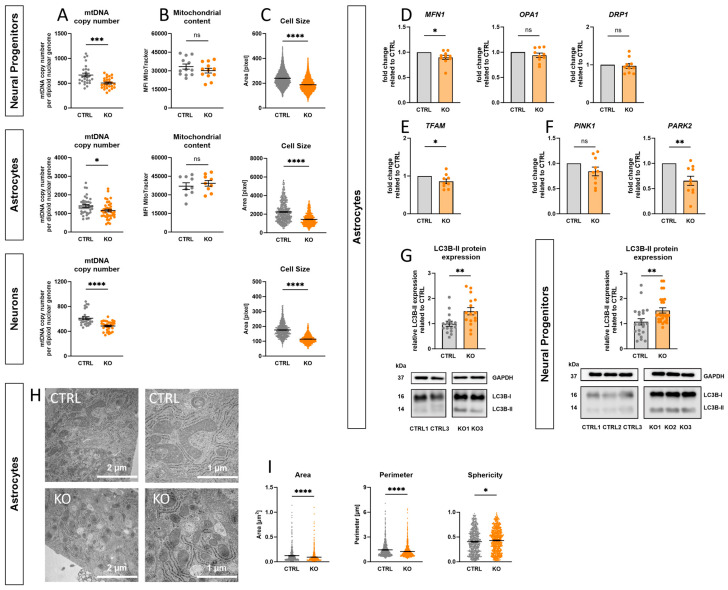
(**A**) mtDNA copy number, (**B**) mitochondrial content, and (**C**) cell size in TSPO knockout and control cells. (**A**) mtDNA copy numbers in TSPO KO and CTRL cells are presented as single values of a minimum of three individual qRT-PCR of *n* = 9 (NPCs), *n* = 6 (astrocytes and neurons) biological replicates. (**B**) Mitochondrial content was measured by flow cytometry and is indicated by MitoTracker Green mean fluorescence intensity (MFI) of *n* = 4 individual experiments; 1 × 10^5^ and 2 × 10^4^ events were recorded for each replicate of NPCs and astrocytes, respectively. (**C**) Cell size was analysed by assessing the area [pixels] of Fura-2/AM-loaded cells, and the dot plots show the number of pixels. Data are presented as mean ± SEM using independent samples *t*-test or Mann–Whitney U test. (**D**–**G**) Relative gene and protein expression in TSPO knockout and control cells. (**D**) Gene expression of mitochondrial fusion and fission proteins *MFN1*, *OPA1*, and *DRP1*; (**E**) mitochondrial transcription factor *TFAM*; and (**F**) mitophagy proteins *PINK1* and *PARK2*. mRNA levels of TSPO-deficient cells related to TSPO-expressing control cells. *MFN1*: 0.9097, *p* = 0.0348; *OPA1*: 0.9393, *p* = 0.1989; *DRP1*: 0.9703, *p* = 0.6444; *TFAM*: 0.8581, *p* = 0.05; *PINK1*: 0.8401, *p* = 0.0988; *Parkin*: 0.6546, *p* = 0.0041. Data are presented as mean single values of five qRT-PCR ± SEM (*n* = 10), Welch’s corrected *t*-test. (**G**) Deletion of TSPO led to increased expression of the lipidated form of LC3B protein (LC3B-II) in hiPSC-derived NPCs and astrocytes. Representative Western blots show the expression of housekeeper gene GAPDH at 37 kDa and LC3B-I at 16 kDa and LC3B-II at 14 kDa. Bar graphs show densitometric, relative mean of LC3B-II expression levels. Individual values were normalised to the respective expression levels of GAPDH and were related to the CTRL mean. Data are presented as mean ± SEM using Mann–Whitney U test (Astro) and independent samples *t*-test (NPCs) of three biological replicates. (**H**,**I**) Analysis of the mitochondrial structure by electron microscopy in TSPO knockout and control astrocytes. (**H**) Representative images of mitochondria from astrocytes of both control and knockout groups (*n* = 3 each). (**I**) Semi-quantitative analysis of mitochondrial area, perimeter, and sphericity. Dot plots display single values of TSPO-expressing (*n* = 686) and TSPO-devoid (*n* = 854) mitochondria. Data are presented as mean ± SEM using Mann–Whitney U test. Asterisks in the figure represent *p*-values as follows: * *p* ≤ 0.05, ** *p* < 0.01, *** *p* < 0.001, and **** *p* < 0.0001.

Consistent with the observation of altered bioenergetic properties and oxidative stress, TSPO-deficient NPCs, astrocytes, and neurons contained significantly less mtDNA compared to their controls (NPC *p* < 0.0001; Astro *p* = 0.017; Neuron *p* < 0.0001) (Figure 7A).

Reduced mitochondrial mass is an adaptive response to decreased mtDNA copy number. Consequently, the mitochondrial content was determined by flow cytometry using the fluorescent dye MitoTracker Green. However, MTG fluorescence did not differ significantly between KO and CTRL cells (NPC *p* = 0.26; Astro *p* = 0.67) (Figure 7B), indicating no effect of TSPO expression on the mitochondrial mass in our cells.

Interestingly, consistent among all cell types examined, cells lacking TSPO protein exhibited a considerably reduced soma size (*p* < 0.0001), as determined by pixel counting of the fluorescent area during Fura-2 imaging (Figure 7C).

### 2.12. Impact of TSPO on Mitochondrial Dynamics and Morphology

Dynamic fusion and fission processes help to adapt the cellular mitochondrial network to cellular demands and external stressors. In addition, mitophagy selectively eliminates damaged or dysfunctional mitochondria, thereby preventing detrimental effects on cellular health.

To investigate the impact of TSPO deficiency on mitochondrial dynamics, the mRNA expression levels of key fusion proteins, mitofusin 1 (*MFN1*) and optic atrophy protein 1 (*OPA1*), and fission protein dynamin-related protein 1 (*DRP1*) were analysed in hiPSC-derived astrocytes. Figure 7D shows that TSPO deficiency significantly reduced *MFN1* mRNA levels (*p* = 0.035), whereas *OPA1* and *DRP1* transcripts remained unchanged. Additionally, the gene expression of mitochondrial transcription factor A (*TFAM*), a crucial activator of mitochondrial transcription and regulator of mtDNA copy number, was examined. Consistent with the decreased mtDNA copy number, *TFAM* mRNA expression was significantly reduced in astrocytes lacking TSPO protein (*p* = 0.05) (Figure 7E). Furthermore, the role of TSPO in mitophagy was examined by gene and protein expression analyses. While PTEN-induced putative protein kinase 1 (*PINK1*) transcripts were not significantly different between TSPO KO and CTRL astrocytes, parkin (*PARK2*) mRNA levels were considerably lower in TSPO-deficient astrocytes (*p* = 0.004) (Figure 7F). Surprisingly, LC3B-II protein expression, the cleaved lipidated form of LC3B, which is tightly associated with autophagosomal membranes and indicates the degree of autophagic activation, was upregulated in TSPO-deficient NPCs and astrocytes. Western blot analyses with an anti-LC3B antibody showed that TSPO deficiency in hiPSC-derived NPCs and astrocytes led to an increase of 42.1 ± 10.3% and 49.9 ± 14.3% in LC3B-II protein expression, compared to the respective control (NPC *p* = 0.007; Astro *p* = 0.0066), as shown in Figure 7G. These findings suggest a role for TSPO in modulating mitochondrial dynamics, mitophagy, and the maintenance of the mitochondrial genome.

As mitochondria morphology is regulated by the dynamic interplay between fusion and fission, ensuring mitochondrial network integrity and function, electron micrographs of hiPSC-derived astrocytes were quantified to investigate the influence of TSPO expression on mitochondrial morphology. Mitochondria devoid of TSPO were significantly smaller, as indicated by a decreased surface area (*p* < 0.0001) and perimeter (*p* < 0.0001). Additionally, the sphericity of the mitochondria was significantly increased (*p* = 0.03) in TSPO KO astrocytes, indicating a rounder, less polymorphic shape, as depicted in Figure 7H,I.

### 2.13. TSPO Expression in Major Depressive Disorder

**Figure 8:**
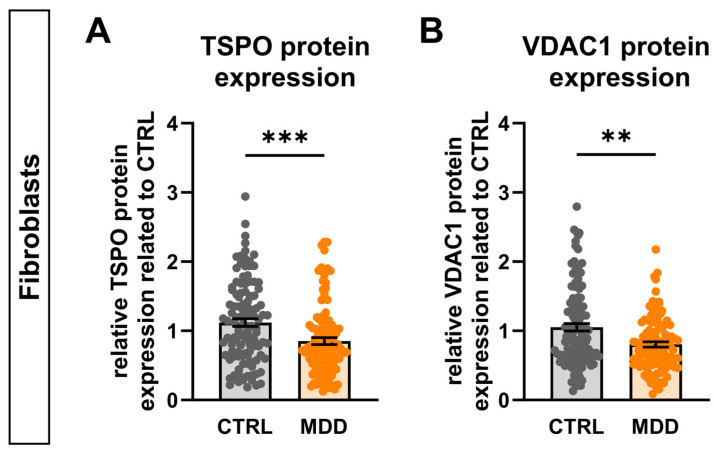
TSPO and VDAC1 protein expression in human primary fibroblasts of major depressive disorder (MDD) patients and healthy controls. Relative expression of (**A**) TSPO and (**B**) VDAC1 proteins normalised to the respective expression levels of BTUB1 and related to the CTRL mean. Bar graphs show densitometric, relative mean ± SEM using Mann–Whitney U test of three individual biological replicates of *n* = 16 fibroblast cell lines. Asterisks in the figure represent *p*-values as follows: ** *p* < 0.01, *** *p* < 0.001.

TSPO has been implicated in neurodegenerative and neuroinflammatory processes, as presumed by the observation of increased TSPO expression in various neuropathologies such as Alzheimer’s disease, multiple sclerosis, Parkinson’s disease, and major depressive disorder (MDD) [12,15]. Although the molecular pathomechanisms underlying MDD are still not fully understood, accumulating evidence suggests a link between mitochondrial dysfunction, bioenergetic imbalance, and the etiology and pathology of depression [26,27,28,39,40,41]. Using a human cell model of depression, our group found mitochondrial function to be altered in a way similar to what we observed in TSPO-deficient cells [27,28]. This possible connection prompted us to analyse the protein expression of TSPO and VDAC1 in cells from patients with MDD. Therefore, we used fibroblast cell lines derived from 16 patients with MDD and their sex- and age-matched controls. Interestingly, we found that the levels of both TSPO (Figure 8A) and VDAC1 (Figure 8B) proteins were significantly reduced (TSPO 76.03 ± 4.94%, *p* = 0.0004; VDAC1 76.48 ± 3.92% *p* = 0.0028) in patients with MDD, pointing towards a potential involvement of TSPO in the development or cause of mitochondrial dysfunction and manifestation of MDD.

## 3. Discussion

In the present study, the effect of TSPO on cellular and mitochondrial homeostasis was investigated using a novel CRISPR/Cas9-mediated TSPO knockout model in human iPSCs. As the physiological significance of TSPO seems to be tissue- and cell-type-specific, TSPO KO and CTRL hiPSCs were differentiated into neural progenitor cells, astrocytes, and neurons to elucidate metabolic changes related to neurosteroid synthesis, cellular and mitochondrial bioenergetics, VDAC1 protein expression, redox and calcium homeostasis, as well as mitochondrial dynamics and morphology in the presence and absence of TSPO across different cell types.

### 3.1. Role of TSPO in Neurosteroidogenesis

TSPO has long been considered essential for mitochondrial cholesterol import. Studies using biochemical, pharmacological, and genetic experimental approaches have provided convincing evidence for the important role of TSPO in steroid production [3,42,43,44,45,46]. However, recent in vivo and in vitro TSPO knockout studies have refuted the role of TSPO in steroidogenesis [47,48,49]; therefore, its specific role in neurosteroid synthesis is still a matter of debate [50].

We found a significant decrease in pregnenolone levels in TSPO-deficient astrocytes, supporting an important role of TSPO in neurosteroid synthesis. However, the amount of detected pregnenolone was considerably low, ranging between 2.5 and 7.2 pg/µg/mL of protein. These low levels indicate a low steroid-synthesising capacity of hiPSC-derived astrocytes, similar to what we found in human C20 microglia cells [7]. Angeloni et al. also suggested a role of TSPO in cholesterol trafficking and neurosteroid synthesis in human microglia, since TSPO silencing resulted in impaired cholesterol homeostasis, leading to excessive cholesterol accumulation [51].

### 3.2. Impact of TSPO on Mitochondrial and Cellular Respiration

TSPO-deficient NPCs and astrocytes showed reduced mitochondrial respiration, as indicated by lower basal and maximal respiration rates. While the cellular ATP contents were not different, the mitochondrial and total ATP production rates were significantly reduced in TSPO KO NPCs and astrocytes, indicating a decreased metabolic flux. Testing the glycolytic activity of TSPO KO cells, which could compensate for decreased OXPHOS and maintain total intracellular ATP levels, revealed a reduced basal and compensatory glycolysis in TSPO-deficient NPCs and astrocytes.

In the human C20 microglial cell line, basal and maximal respiration, as well as ATP-related oxygen consumption, were significantly reduced in TSPO knockout cells compared to wild-type cells. Lentiviral overexpression of TSPO was able to rescue the impaired mitochondrial respiration in C20 knockout cells [7].

Decreased mitochondrial respiration could result from lower activity in the mitochondrial respiratory chain or a shortage of substrates or enzymes. If the availability of glucose or fatty acid substrates is limited, or if the enzymes responsible for their metabolic breakdown are impaired, the levels of NADH/H+ and FADH2 reduction equivalents would decline. Additionally, the transport of these reduction equivalents into the mitochondria might also be a limiting factor for the function of the electron transport chain [52].

The voltage-dependent anion channel VDAC is a crucial regulator of multiple mitochondrial functions. As the major pore of the OMM, VDAC1 facilitates the translocation of respiratory chain substrates, such as ADP, ATP, NAD^+^, and NADH. Furthermore, VDAC is involved in the regulation of metabolite diffusion, including glucose, pyruvate, glutamate, succinate, and citrate and cations, such as Ca^2+^ and Mg^2+^ [53]. The association between TSPO and VDAC has been extensively studied, suggesting their involvement in an insufficiently characterised protein complex that exerts a joint influence on physiological functions.

TSPO deficiency was accompanied by reduced VDAC1 protein expression in hiPSC-derived NPCs and astrocytes. While the gene expression level remained constant, protein stability assays conducted with cycloheximide revealed increased degradation of VDAC1 protein in TSPO KO NPCs. This negative correlation between TSPO and VDAC expression has also been shown in a TSPO knockout model of human C20 microglial cells [7,8]. A mutual dependence between TSPO and VDAC expression is also demonstrated in patients with bipolar disorder who exhibited significantly increased expression levels of both TSPO and VDAC in their peripheral blood mononuclear cells (PBMCs) [54].

Overall, the reduced mitochondrial and cellular respiration observed in TSPO-deficient cells can be caused by various factors, including diminished ETC activity, restricted substrate or enzyme availability, malfunctioning of ETC complexes, and improper functioning of the TSPO-VDAC interactome. Further investigations are needed to fully understand the mechanisms underlying the regulatory role of TSPO in cellular and mitochondrial respiration.

### 3.3. Mitochondrial Membrane Potential and Ca^2+^ and Redox Homeostasis

A more negative or polarised MMP implies an increase in the coupling efficiency of OXPHOS, whereas a less negative or depolarised MMP suggests a decrease in efficiency or may result from rate limitations in the ETC or substrate oxidation [55]. Consistent across all analysed cell types in our study, TSPO deficiency resulted in a reduction in the MMP. This is in line with several studies reporting that inhibition of TSPO expression reduced the membrane potential of the mitochondria. In murine BV-2 microglial cells, RNA interference-mediated TSPO knockdown resulted in lower MMP [14,56]. Similarly, mitochondrial depolarisation has been observed in primary microglia isolated from genetically modified TSPO knockout mice [56,57]. Moreover, CRISPR/Cas9-mediated knockout of TSPO in human C20 microglia caused a robust reduction in the MMP [7].

Consistently, all TSPO knockout cells showed decreased cytosolic and increased mitochondrial Ca^2+^ levels. This is in line with a previous study that reported an increase in [Ca^2+^]_m_ and a decrease in [Ca^2+^]_c_ in TSPO knockdown cells and reversed effects in TSPO-overexpressing cells from different species, suggesting a conserved mechanism of mitochondrial Ca^2+^ homeostasis. The proposed mechanism involves TSPO, which limits the transport efficiency of Ca^2+^ ions into the mitochondria and controls mitochondrial Ca^2+^ uptake through VDAC [11].

Alterations in cellular Ca^2+^ signalling cascades, such as altered Ca^2+^ release from intracellular stores, impaired Ca^2+^ sensing and buffering mechanisms, and dysregulation of Ca^2+^-transporting proteins or channels, can affect mitochondrial function and compromise OXPHOS. Although Ca^2+^ is required for ATP synthesis, an excess influx of Ca^2+^ into the mitochondrial matrix can lead to increased ROS formation, which eventually induces MMP loss and may trigger mPTP opening, ultimately resulting in apoptosis [58]. While low levels of ROS serve crucial signalling functions, excessive ROS accumulation leads to oxidative stress and molecular damage. Flow cytometry analysis of hiPSC-derived NPCs and astrocytes revealed a significant increase in cytosolic ROS and mitochondrial superoxide levels in TSPO-deficient cells. Similarly, TSPO knockout murine glioma cells and TSPO knockdown human microglial cells displayed significantly higher cytosolic ROS levels than wild-type cells [51,59].

Furthermore, Gatliff et al. reported that dysregulation of mitochondrial Ca^2+^ signalling and the subsequent parallel increase in intracellular [Ca^2+^]_c_ in TSPO-overexpressing cells resulted in elevated mitochondrial ROS levels following the activation of Ca^2+^-dependent NADPH oxidase [11].

### 3.4. Involvement of TSPO in Mitochondrial Dynamics and Mitophagy

In accordance with the observed impaired bioenergetic status, mtDNA copy numbers were significantly reduced in TSPO knockout cells across all analysed cell types. Consistently, human TSPO KO C20 microglial cells contained significantly less mtDNA than control cells [8]. In contrast, Yao et al. demonstrated that although TSPO knockdown in BV-2 microglia resulted in decreased MMP and OXPHOS, the mtDNA copy number increased in addition to a higher amount of released mtDNA, indicating IMM damage [56].

Nuclear-encoded mitochondrial transcription factor A (TFAM) serves as a key activator of both transcription and replication and plays a crucial role in regulating mtDNA copy number [60]. TSPO-deficient hiPSC-derived astrocytes exhibited significantly lower levels of *TFAM* transcripts. TFAM knockdown cell models with reduced mtDNA copy numbers showed downregulation of mitochondrial transcription, lower respiratory enzyme activity, and reduced expression of mtDNA-coded complex proteins involved in OXPHOS [61].

TSPO knockout hiPSC-derived astrocytes contained significantly lower *MFN1* mRNA levels than CTRL cells, whereas *OPA1* and *DRP1* transcripts were unaltered. TSPO-deficient mouse glioma cells also showed a marked reduction in MFN1 levels and no change in DRP1 protein expression. However, the expression of mitochondrial fission proteins MFF and FIS1 as well as of the fusion proteins MFN2 and OPA was increased in TSPO-deficient cells [59].

Although only the mitochondrial fusion gene *MFN1* was affected in TSPO knockout astrocytes, mitochondria devoid of TSPO protein were significantly smaller and rounder. TSPO-silenced mitochondria from hepatocellular carcinoma cells were also found to have smaller mitochondria with fewer cristae [62]. Similarly, TSPO knockout in mouse GL261 glioma cells resulted in mitochondrial fragmentation, whereas wild-type cells contained more fused or elongated mitochondria [59]. In contrast, an analysis of mitochondrial morphology in human SH-SY5Y and canine CF35 cells revealed a more elongated and branched mitochondrial network in TSPO knockdown cells [11,63].

LC3B-II protein expression, which is tightly associated with the autophagosomal membrane and indicates the degree of autophagic activation, was upregulated in TSPO-deficient NPCs and astrocytes. Gatliff et al. proposed an inhibitory function of TSPO via VDAC1 by limiting parkin-dependent ubiquitination. TSPO, VDAC1, and parkin may collectively serve as molecular platforms for regulating the autophagosome-mediated removal of mitochondria, depending on the homeostatic expression ratio of these proteins [10]. Thus, reduced VDAC expression caused by TSPO deficiency may lead to increased autophagy through ubiquitination and degradation of mitochondrial proteins. It has been observed that cells expressing only low levels of TSPO show upregulation of mitochondrial ubiquitination and increased mitophagy, which is associated with a depolarisation of the MMP [10,11]. Treatment with the mitochondrial uncoupler FCCP, which is commonly used to activate PINK1/parkin-mediated mitophagy upon MMP depolarisation, further increased the mitophagy index. In the same line of evidence, TSPO overexpression reduced mitophagy [63].

Therefore, our findings indicate that mitochondrial dysfunction induced by TSPO depletion can trigger the elimination of malfunctioning mitochondria, linking TSPO function to mitochondrial dynamics, mtDNA maintenance, and mitophagy.

TSPO knockout NPCs, astrocytes, and neurons not only displayed impaired mitochondrial function but also exhibited a significant reduction in cellular size, which was also recently reported for human TSPO-deficient and -silenced C20 microglial cells [8,51]. Typically, cellular organelles and protein content scale isometrically with cell size. However, mitochondrial activity, i.e., MMP and OXPHOS, is highest in cells of intermediate size. This nonlinearity of mitochondrial functionality results in an optimal cell size to maximise cellular fitness and proliferation capacity [64]. It has been shown that maintaining optimal cell size and scaling of mitochondrial functions depends on the normal morphology and dynamics of the mitochondria. The mevalonate pathway, which synthesizes plasma membrane components including cholesterol, affects mitochondrial dynamics and functionality scaling [64]. According to a study by Mourier et al., mitofusin knockout leads to decreased protein and metabolite levels in the mevalonate pathway [65]. Given the reduced *MFN1* gene expression and apparent increased mitochondrial fragmentation observed in TSPO KO cells, TSPO-deficient cells appear to lose the ability to maintain their optimal cell size. However, the putative involvement of impaired mevalonate metabolism requires further investigation.

### 3.5. Possible Role for TSPO in Mitochondrial Dysfunction in Depression

Mitochondrial function has emerged as an important factor in the pathophysiology of major depressive disorder (MDD). Reduced bioenergetic capability, oxidative stress, and impaired mitochondrial function render cells vulnerable to stress and may contribute to the development of MDD and other psychiatric disorders [40,66,67]. Malfunctioning mitochondria-related effects are not limited to neuronal cells, but have been reported in peripheral non-neuronal cells, such as fibroblasts [28,41], muscles [68], PBMCs [39], and platelets [69,70] of depressed patients, highlighting the systemic nature of mitochondrial dysfunction in MDD.

TSPO has already been linked to anxiety and depression disorders, making it a potential diagnostic and therapeutic target. Moreover, TSPO expression is lower in MDD patients receiving antidepressant medication than in unmedicated patients [71], and TSPO binding is greater in patients with chronologically advanced MDD and a long duration of untreated depression [72].

Our data demonstrate a significant decrease in the expression levels of both TSPO and the TSPO-interacting protein VDAC1 in fibroblasts obtained from depressed patients. Given the fact that TSPO deficiency in hiPSC-derived neural cells was accompanied by reduced VDAC1 protein levels and led to mitochondrial alterations, altered TSPO and VDAC expression may affect mitochondrial function, neurotransmitter signalling, and neurosteroidogenesis, thereby contributing to the etiology of MDD pathogenesis. Remarkably, the impact of TSPO deficiency on mitochondrial bioenergetics closely resembles parameters of mitochondrial function observed in a human cell model of depression [26,27,28].

The fact that TSPO loss impaired cellular energy metabolism, as evidenced by altered Ca^2+^ homeostasis, reduced OXPHOS, and depolarisation of MMP, is in favour of the hypothesis that TSPO might be a promising target for the treatment of psychiatric disorders. This is also supported by the role of TSPO in the regulation of neurosteroid synthesis. Neurosteroids are important modulators of the GABAergic and glutamatergic systems, which are important targets in the treatment of anxiety and stress-related disorders [15,20,73]. However, correlation does not imply causation and a deeper understanding of the mechanisms behind mitochondrial function and the disruption of mitochondrial bioenergetics in MDD in relation to TSPO expression and function is needed.

## 4. Materials and Methods

### 4.1. Fibroblasts and Human-Induced Pluripotent Stem Cells

Fibroblast cell lines derived from 16 MDD patients and their sex- and age-matched controls [28] were used in this study. The hiPSC line from healthy fibroblasts was generated prior to this study using non-integrating episomal plasmid vectors encoding KLF4, SOX2, L-MYC, LIN28, OCT3/4, and p53DD (pCXB-EBNS, pCE-hsk, pCE-hUL, pCE-hOCT3/4, and pCE-mp53DD (Addgene plasmid #41857, #41814, #41855, #41813, and #41856, gifted by Shinya Yamanaka)) using the Amaxa Nucleofactor (Lonza, Basel, Switzerland). Undifferentiated human hiPSCs were cultured and maintained in mTeSR Plus medium (StemCell Technologies, Köln, Germany) supplemented with 50 µg/mL gentamycin on Matrigel-coated plates (8.7 µg/cm^2^) under standard conditions (37 °C, 5% CO_2_).

### 4.2. C20 Microglia and H295-R Cells

Human microglia C20 cells [8,74] were grown in Dulbecco’s Modified Eagle’s Medium/Nutrient Mixture F-12 Ham (Sigma Aldrich, Taufkirchen, Munich, Germany), supplemented with 10% fetal calf serum (FCS), 2 mM L-glutamine, and 10,000 U/mL penicillinstreptomycin at 37 °C in humidified air with 5% CO_2_, and the medium was changed three times a week.

Adrenocortical carcinoma NCI-H295R cells (CLS, Eppelheim, Germany) were cultured in DMDM/F12 medium containing 15mM HEPES, 6.25 µg/mL insulin, 6.25 µg/mL transferrin, 6.25 ng/mL selenium, 1.25 mg/mL BSA, 5.35 mg/mL linoleic acid, and 2.5% Nu-Serum I, supplemented with 100 U/mL penicillin and 0.1 mg/mL streptomycin, (Thermo Fischer Scientific, Darmstadt, Germany). The cells were maintained in humidified air with 5% CO_2_ at 37 °C, and medium was changed three times a week.

### 4.3. Generation of TSPO Knockout in iPSC Using CRISPR/Cas9 Genome Editing

CRISPR/Cas9-TSPO sgRNA plasmids were prepared according to a previously published protocol [75]. Two independent sgRNA-targeting sequences, #116: 5′-TCCCGCTTTGTCCACGGCGAGGG-3′, and #126: 5′-TCCACGGCGAGGGTCTCCGCTGG-3′ within exon 2 of the human TSPO gene were selected using the bioinformatics tool CRISPOR (http://crispor.tefor.net/, accessed on 27 November 2020). The DNA sequences were separately cloned into the modified pSpCas9 (BB)-2A-Puro (PX459) V2.0 vector (#62988, Addgene, Cambridge, MA, USA), kindly provided by Emmanuel Nivet [76]), using BbsI restriction sites. Constructs were transfected into iPSC cells by nucleofection using human stem cell nucleofector kit 2 (Lonza), and the puromycin-resistant single clones were picked and expanded. Homozygous TSPO KO clones were identified by immunoblotting using an anti-TSPO antibody. To determine if the individual clones were homozygous for TSPO KO, genomic DNA spanning exon 2 was PCR-amplified using the following primers: TSPO-ex2F: 5′-CTGGAAATGCGTTCACTCAG-3′ and TSPO-ex2R: 5′-GCCTGGAGAAGACCCTCTGT-3′, and the PCR product was cloned into the pGEM-T cloning vector (Promega, Walldorf, Germany). Single bacterial colonies of each TSPO KO clone were subsequently sequenced. The top five potential off-target sites, which were selected using the CRISPOR web tool, were likewise PCR amplified, subcloned, and sequenced using specific primers (Supplementary Table S2).

### 4.4. Differentiation of iPSCs into Neural Progenitor Cells (NPCs), Neurons, and Astrocytes

NPCs were derived from iPSCs following a monolayer culture method based on a previously published protocol [31]. For neuronal differentiation, dissociated NPCs from passages 5 to 12 were plated onto laminin-coated µ-dishes (Ibidi, Gräfelfing, Germany) at a density of 1 to 1.5 × 10^5^ cells/cm^2^ and differentiated for 21 days in BrainPhys neuronal medium (StemCell) supplemented with 1% B27 Plus, 0.5% GlutaMax, 0.5% non-essential amino acids, 0.5% Culture One, 200 nM ascorbic acid, 20 ng/mL BDNF, 20 ng/mL GDNF, 1 mM dibutyryl-cAMP, 50 U/mL Penicillin, and 50 μg/mL streptomycin, with half-medium change twice per week. Additionally, 4 µg/mL laminin was freshly added each time the medium was changed.

iPSC-derived NPCs were differentiated into astrocytes, according to a previously described protocol [30]. Briefly, NPCs were plated at 30,000 cells/cm^2^ on Matrigel-coated plates (8.7 µg/cm^2^) and grown for 30 days in astrocyte medium (ScienCell Research Laboratories, Carlsbad, CA, USA). Astrocytes between 31 and 60 days of differentiation were used in this study.

### 4.5. Western Blotting

To block de novo protein biosynthesis and thus determine protein half-life, iPSC-derived NPCs were treated with 100 µg/mL of the translational inhibitor cycloheximide (CHX) for the indicated time points. Whole-cell protein samples were prepared in RIPA buffer, separated by SDS-PAGE, and transferred onto a nitrocellulose membrane. Blotted proteins were detected using anti-TSPO (ab109497) and anti-VDAC1 (ab186321) antibodies, both from Abcam and anti-LC3B (#3868S, Cell Signalling, Danvers, MA, USA). Anti-Beta 1 tubulin (clone E7, from DSHB, Iowa City, IA, USA) and anti-GAPDH (sc-47724, Santa Cruz Biotechnology, Heidelberg, Germany) antibodies were used as loading controls. Densitometric analysis was performed using ImageJ software (version 2.9.2).

### 4.6. Immunofluorescence

For immunocytochemistry, cells grown on Geltrex-, Matrigel-, or laminin-coated glass coverslips (12 mm) or µ-dishes (Ibidi) were fixed in 4% PFA. Cells were permeabilised and blocked using a blocking solution containing 1× PBS, 0.5% Triton X-100, and 10% normal goat serum. Antigen retrieval was performed prior to staining of TSPO and VDAC1 by steaming coverslips in citrate buffer, pH 6. Cells were incubated overnight at 4 °C with following primary antibodies: anti-ALDHL1 (ab87117; 1:500), EAAT1 (ab416-1001; 1:200), anti-MAP2 (ab5392; 1:500), anti-NeuN (ab177487; 1:1000), anti-TSPO (ab109497; 1:500), anti-SYP (ab52636; 1:250), anti-VDAC1 (ab186321; 1:500), and anti-VGLUT1 (ab180188; 1:250), all from Abcam. Anti-GFAP (C53893; 1:400) and anti-S100β (S2532; 1:1000) were purchased from Sigma, and anti-PSD95 (K28/43; 1:250) from NeuroMab (Davis, CA, USA). After three washing steps, cells were incubated for 1 h with corresponding secondary antibodies conjugated with Alexa Fluor 488, Cy3, Cy5, and Hoechst to stain nuclei. Cells were mounted on glass slides with DAKO fluorescent mounting medium and examined using an Axio Observer Z.1 microscope (Zeiss, Jena, Germany).

### 4.7. Fluorescent Live-Cell Imaging

Live-cell imaging experiments were conducted as described in [26]. Briefly, all recordings were taken using an inverted Zeiss Axio Observer Z.1 microscope and detected using an AxioCam MRm CCD camera (Zeiss, Jena, Germany). Illumination control and image acquisition were performed using a Lambda DG-4 high-speed wavelength switcher (Sutter Instruments, Novato, CA, USA) and the ZEN 2012 imaging software. Analysis of the measurements in the regions of interest, manually drawn over selected cells in the visual field, was performed using ImageJ (version 2.9.2). The macros used for background subtraction and analysis are provided upon request.

To analyse mitochondrial membrane potential (MMP) in NPCs and neurons, the ratiometric fluorophore JC-1 (5,5′,6,6′-tetrachloro-1,1′,3,3′-tetraethylbenzimidazolylcarbocyanineiodide) was used. In astrocytes, the fluorescent dyes TMRE (tetramethylrhodamine, ethyl ester) in non-quenching mode and MitoTracker^®^ Green (MTG) were used.

Cytosolic and mitochondrial Ca^2+^ levels were measured using Fura2-AM and Rhod-2AM. Approximately 1.8 × 10^6^ NPCs and 2 × 10^5^ astrocytes were seeded onto Matrigel-coated 25 mm glass coverslips and incubated overnight in a humidified incubator at 37 °C and 5% CO_2_. The following day, the cells were simultaneously loaded with Fura-2/AM and Rhod-2/AM at a final concentration of 2 µM in OptiMEM. Neurons were likewise stained and measured on day 21 of differentiation.

### 4.8. Quantitative Real-Time PCR and mtDNA Copy Number Analysis

For gene expression analysis, total RNA was extracted using RNA Plus Kit (Macherey-Nagel) according to the manufacturer’s instructions. First-strand cDNA synthesis from 1 µg of total RNA was performed with QuantiTect Reverse Transcription Kit (Qiagen, Hilden, Germany). Quantitative real-time PCR was performed on a RotorGeneQ (Qiagen) machine using the Takyon SYBR mastermix (Eurogentec, Köln, Germany) and specific intron-spanning primers, listed in Supplementary Table S3. Measurements were performed in technical duplicates with a minimum of three biological replicates. The results were analysed using RotorGeneQ software V2.3 (Qiagen) by applying the ΔΔCt method for relative quantification.

To quantify the mtDNA copy number, primers for the mitochondrial-encoded tRNA leucine 1 (mt-TL1) and the nuclear gene Beta-2 microtubulin (B2M) were used. Instead of total RNA, genomic DNA was used, and the following equation for quantification of mtDNA content relative to the diploid nuclear DNA was applied:

mtDNA copy number = 2 × E^(−∆∆Ct)^ (1)

### 4.9. Mitochondrial Respirometry

A total of 80,000 NPCs/well or 30,000 astrocytes/well were grown on a Matrigel-coated XFp miniplate (Agilent, Santa Clara, CA, USA). The sensor cartridges were prepared according to the manufacturer’s recommendations. To test oxidative phosphorylation, the sensor plate was loaded with oligomycin (1 µM), FCCP (1 µM for NPCs and 2 µM for astrocytes), and a combination of rotenone (0.5 µM) and antimycin A (0.5 µM). For measuring the glycolytic rate by calculating and subtracting mitochondrial-produced acidification, the mitochondrial inhibitors rotenone and antimycin A were used in a final concentration of 0.5 µM each as well as 50 mM 2-deoxy-D-glucose (2-DG) as a glycolytic inhibitor.

The total ATP production rate was measured by obtaining both OCR and ECAR, simultaneously under basal conditions and after serial addition of 1.5 µM oligomycin followed by 0.5 µM of each rotenone and antimycin A. For the normalisation of respiration data, cells were stained with Hoechst and nuclei were counted using a custom made ImageJ macro (provided on request).

### 4.10. Total ATP Content Quantification

For quantification of whole-cell ATP content, the CellTiter-Glo^®^ Luminescent Cell Viability Kit (Promega) based on an ATP-dependent luciferase reaction was used. ATP was measured in cell pellets of 1 × 10^6^ NPCs or 1 × 10^5^ astrocytes according to the manufacturer’s instructions. ATP concentrations were normalised to µg/mL protein using a BCA assay (Thermo Fisher Scientific).

### 4.11. Scanning Electron Microscopy (SEM)

A detailed procedure has been described previously [77]. Briefly, iPS neurons grown on glass slides were viewed in high-vacuum (high-vac) SEM mode. Cells were fixed for 30 min at room temperature with 2.5% glutaraldehyde (Serva, Heidelberg, Germany) in 0.1 M Soerensen buffer, pH 7.4 (Merck, Darmstadt, Germany), and aqua bidest. After treatment with the critical point dryer Balzers CPD 030 (BAL-TEC AG, Pfäffikon, Switzerland; Leica, Wetzlar, Germany ) and positioning on stubs via Leit-Tabs (Science Services, Munich, Germany), cells were sputtered with platinum twice for 30 s at 30 mA at a distance of 50 mm (BAL-TEC SCD 005, BAL-TEC AG, Leica). Cells were kept in the exicator and investigated under dry conditions with an FEI Quanta 400 FEG in high-vac mode (4.0 kV, Spot 3, WD ≈ 6 mm, ≤10^−4^ Torr, tilt 30°).

### 4.12. Pregnenolone Quantification

Pregnenolone quantification was performed using an enzyme-linked immunosorbent assay (ELISA), adapted from a previously published protocol [78]. Briefly, pregnenolone concentration was measured in the supernatant from 1.5 × 10^5^ astrocytes/well of a Matrigel-coated 24-well plate. To quantify pregnenolone accumulation, the supernatant was collected after 3 h of incubation at 37 °C in ELISA buffer (140 mM NaCl, 5 mM KCl, 1.8 mM CaCl_2_, 1 mM MgSO_4_, 10 mM glucose, 10 mM (HEPES)-NaOH, pH 7.4, and 0.1% BSA) containing inhibitors of pregnenolone metabolism trilostane (25 µM) and SU10603 (10 µM) using pregnenolone ELISA (LDN, Nordhorn, Germany) according to the manufacturer’s instructions. Quantified pregnenolone concentrations were normalised to the protein concentration of each sample, as determined using the Pierce BCA Protein Assay (Thermo Fisher Scientific, Waltham, MA, USA).

### 4.13. Flow Cytometry Analyses

Flow cytometry was performed on a BD Biosciences FACS Celesta™, which was run using Diva software v7.0 (BD Biosciences, Franklin Lakes NJ, USA). The cytometer was equipped with violet (405 nm), blue (488 nm), and red (640 nm) lasers. Particle size and granularity were measured using forward scatter (FSC) and side scatter (SSC) detectors. The area, height, and width parameters of the FSC and SSC detectors were used for doublet exclusion. Compensation was not required. For each sample, 0.15 × 10^6^ to 0.3 × 10^6^ (astrocytes) or 0.5 × 10^6^ to 1 × 10^6^ (NPCs) cells were used. All tests were performed in at least three independent experiments. Data were acquired immediately after staining using an FACS Celesta™ flow cytometer (BD Biosciences). FlowJo software (V10.8, Tree Star, Ashland, OR, USA) was used for analysis.

### 4.14. Cytosolic and Mitochondrial Reactive Oxygen Species/Oxidative Stress

Cytosolic ROS levels were determined by applying 10 µM 2′,7′-dichlorofluorescein diacetate (H2DCFDA) for 20 min under standard culture conditions in FACS buffer (DPBS supplemented with 2% FBS) in airtight tubes. To analyse mitochondrial ROS production, cells were washed with Hank’s Balanced Salt Solution (HBSS), stained with 5 µM MitoSOX^TM^ Red Superoxide indicator in HBSS, and incubated in a humidified atmosphere at 37 °C and 5% CO_2_. Flow cytometry data were acquired using the Fortessa System (BD) or Celesta System (BD). Data were analysed using FlowJo (v10.8.1).

### 4.15. Mitochondrial Mass

Mitochondrial content was assessed by staining with 1 µM MitoTracker^®^ Green in RPMI 1640 supplemented with 2 mM L-glutamine for 1 h at 37 °C in a cell incubator. To prevent mitochondrial depolarisation, 1.5 µM cyclosporin A was added. After washing and resuspension in 200–300 µL FACS buffer, the cells were filtered and immediately analysed by flow cytometry.

### 4.16. Statistical Analysis

Graphical depictions and statistical analyses were performed using GraphPad Prism 9.5.1 (GraphPad Software, Boston, MA, USA) for at least three independent experiments. For all experiments, two to three technical replicates were calculated and at least three biological replicates were averaged. Statistical outliers were identified using the ROUT method and removed before the analysis. The normal distribution of the data was assessed using the D’Agostino-Pearson omnibus normality test. Nonparametric data that did not follow a Gaussian distribution were analysed using the Mann–Whitney U test, whereas parametric data were analysed using an independent samples *t*-test. The cut-off value for statistical significance was set at *p* ≤ 0.05. Results are presented as mean ± standard error of the mean (SEM). Asterisks in the figures represent *p*-values as follows: * *p* ≤ 0.05, ** *p* < 0.01, *** *p* < 0.001, and **** *p* < 0.0001.

## 5. Conclusions

The comprehensive functional investigation of our human neuronal cell model revealed significant mitochondrial dysfunction in TSPO-deficient cells, which encompassed various mutually dependent aspects of mitochondrial biology. The reduced pregnenolone synthesis observed in TSPO knockout astrocytes confirms TSPO’s contribution to the production of neurosteroids, such as progesterone and allopregnanolone, which are important modulators of neuronal activity and neurotransmission, necessary for normal brain function. The impaired cellular and mitochondrial bioenergetics in TSPO-deficient cells indicate the importance of TSPO in sustaining the energy-producing capacity of mitochondria. Reduced OXPHOS and glycolysis suggest disruptions in the metabolic processes necessary for cellular energy supply. The altered mitochondrial morphology and dynamics observed in TSPO KO astrocytes suggest the involvement of TSPO in regulating the dynamic structural integrity and functional organisation of mitochondria, which is important for preserving mitochondrial health and functionality. Moreover, increased mitophagy and a subsequent decrease in mtDNA copy number in TSPO KO cells indicate a disturbance in the normal turnover and maintenance of mitochondria. This suggests that TSPO may be involved in modulating mitochondrial quality control. Overall, these findings highlight the intricate effects of TSPO on the maintenance of mitochondrial integrity and function.

The precise molecular mechanisms underlying the effects of TSPO, and the context-dependent nature of its action remain widely elusive. However, the interaction of TSPO with other mitochondrial and cellular proteins, forming complex molecular networks, appears to be crucial for its function. The composition of these functional complexes remains a subject of ongoing research. VDAC is the major pore in the OMM and serves as a critical pathway for the import and export of metabolites to and from the mitochondria, playing a crucial role in mitochondrial homeostasis. A reduced VDAC expression in TSPO knockout cells indicates a possible interaction between TSPO and VDAC and establishes a functional link between TSPO- and VDAC-associated functions. This correlation may contribute to the mechanism of action of TSPO in mitochondrial physiology.

The observed decrease in TSPO and VDAC protein expression in a human cell model of depression raises intriguing possibilities regarding the role of TSPO in the pathophysiology of stress-related disorders and depression. Further research on the function of TSPO and its physiologically relevant interaction partners will contribute to a deeper understanding of its role in maintaining cellular homeostasis. Moreover, these investigations may uncover new therapeutic targets for mitochondria-related disorders.

## Data Availability

Data generated in this study are available from the corresponding author and will be provided upon reasonable request.

## Supplementary Materials

The following supporting information can be downloaded at: https://www.mdpi.com/article/10.3390/ijms252312882/s1.
