# Supplementary material for: A Comprehensive Functional Investigation of the Human Translocator Protein 18 kDa (TSPO) in a Novel Human Neuronal Cell Knockout Model
Source: Int J Mol Sci. 2024 Nov 29;25(23):12882. doi: 10.3390/ijms252312882 (PMC11641651; doi:10.3390/ijms252312882)
Supplement: Supplementary file 1 [file ijms-25-12882-s001.zip › ijms-3317308-supplementary.pdf]

Supplementary Table S1: Generation of PAX6/SOX2 positive hiPSC-derived NPC. Numbers represent proportion [%] of SOX2 and PAX6 positive cells in the analysed cultures.

Cell line	SOX2 positive [%]	PAX6 positive [%]
CTRL1	89.7	93.0
CTRL2	91.6	95.2
CTRL3	88.8	91.1
KO1	88.7	94.7
KO2	93.8	95.9
KO3	87.1	93.2

Supplementary Table S2: Primer sequences used for off-target screening

Off-Target	5‘3‘-Primer Sequence	
116-OFF#1	forward	CGCCTACCTCAATACACCCT
	reverse	CACCCTACTAGCTAGTCCCG
116-OFF#2	forward	TAAGCTGCCTCTTACCCTGC
	reverse	GAGTCTTCAGGGCCTCACAT
116-OFF#3	forward	GTGGCTTACAAACTGGGCTC
	reverse	CAGCGGGCATATACCTCTCT
116-OFF#4	forward	CTCTTGGCTGTCCTGTCTCA
	reverse	GTGCCTGTTGTTGTGTGGAT
116-OFF#5	forward	GCTCCTTTCCACCTTCCTCT
	reverse	CTGCGTTCAAGAAATGGCCT
126-OFF#1	forward	CCCTGCCTGCTAAATCCAAT
	reverse	ATTGAGACTGTCGACGGAGG
126-OFF#2	forward	TGTGTGAAGGACGAGGCTTT
	reverse	GTCTCGATCTCCTGACCTCG
126-OFF#3	forward	CCAGACTCAACCTCAGCTGA
	reverse	AGAAAGCTCTGGGGTCAAGA
126-OFF#4	forward	AATGATTACCGGGGCTTCCT
	reverse	ATGTAGTTGGCTGGCAGTCT
126-OFF#5	forward	TCCCAGGTTCAAGCGATTCT
	reverse	TTCCTCAAAGATCCCAGGGG

CRISPR/Cas9 genome editing can lead to undesired editing outcomes, both on-target at the intended editing site and off-target at other genomic loci. To ensure the reliability of the results and eliminate the possibility of biased outcomes due to potential off-target effects, three independent isogenic clones were created. Additionally, three distinct isogenic control hiPSC lines were generated as a physiological reference.

To allow valid comparisons between edited cells and their non-edited isogenic controls, unintended alterations must be excluded. Therefore, guide RNAs with low predicted off-target scores were selected using the CRISPOR web tool. After genome editing, potential off-target sites were analysed by DNA sequencing of the top five off-target hits from the scoring algorithm. The top five off-target candidates within the genome remained unaltered after CRISPR/Cas9 editing using sgRNAs #116 and #126.

Off-target screening

sgRNA	Off-target Sequence	Locus description	Sequencing
#116	TCCTGTTTAGTCCACGGCAAAGG	chr9<br>intron: CTSV	✓
	TCCTGCTTTATCCACAGAGATGG	chrX<br>intergenic: LL0XNC01-73E8.1-BEX1	✓
	TGCAGCTCTGTCCACGGTGAGGG	chr8<br>intron:DNAJC5B	✓
	TTCCTTTTTGTCCACAGCGAGGG	chr9<br>intron:RAPGEF1	✓
	TCCCTCTCAGTCCACGGCAAAGG	chr18<br>intergenic:Y_RNA-RP11-715C4.1	✓
#126	TTCTCAGCGAGGGTCTCCTCTGG	chr20<br>intergenic:RP5-984P4.4-PAX1	✓
	TCCATGGTGAGGATCTCTGCAGG	chr19<br>exon:SPC24	✓
	TCCACAGTCAGGGTCTCCTCTGG	chr1<br>exon:SH3D21	✓
	GCCACCGCTAGGGTCTCCACAGG	chrX<br>exon:FAM127B	✓
	GCCACGGGCAGGGTCCCCGCAGG	chr7<br>intergenic:AC091729.9-UNCX	✓

Supplementary Table S3: Primer sequences used for gene expression analysis

Gene	5'3'-Primer Sequence	
HPRT	forward	TTGCTTTCCTTGGTCAGGCA
	reverse	ATCCAACACTTCGTGGGGTC
DRP1	forward	TCAAGACAGTGTGCCAAAGG
	reverse	TGGCCTACTAGCTCACTCTGA
MFN1	forward	AGGATGATTGTTAGCTCCACGA
	reverse	CACAGGCGAGCAAAAGTGG
OP1	forward	ACTGAAGTTAGGCGATTAGAGAA
	reverse	CCAGTTGAACGCGTTTACCA
Parkin	forward	AGAAAACCACCAAGCCCTGT
	reverse	CAGTTCCAGCACCACTCGAG
PINK1	forward	GTACCAGTGCACCAGGAGAA
	reverse	AACCTGCCGAGATGTTCCAC
TFAM	forward	CGCAGGAAAAGCTGAAGACTG
	reverse	TCGTCCTCTTTAGCATGCTGA
B2M	forward	TGCTGTCTCCATGTTTGATGTATCT
	reverse	TCTCTGCTCCCCACCTCTAAGT
mt-TL1	forward	CACCCAAGAACAGGGTTTGT
	reverse	TGGCCATGGGTATGTTGTTA

Supplementary Table S4: Cytosolic and mitochondrial calcium concentration in hiPCS-derived NPCs, Astrocytes, and Neurons. (Mann-Whitney U, MW)

	**Neural Progenitors**		**Astrocytes**		**Neurons**	
	CTRL	KO	CTRL	KO	CTRL	KO
**$[Ca^{2+}]_c$** [ratio]	0.590±0.0008<br>n=5380	0.569±0.0005<br>n=5159	0.638±0.001<br>n=1206	0.628±0.001<br>n=1055	1.637±0.02<br>n=910	0.9984±0.01<br>n=1049
	p<0.0001 (MW)		p<0.0001 (MW)		p<0.0001 (MW)	
**$[Ca^{2+}]_m$** [MFI]	156.7±0.88<br>n=4485	163.7±0.88<br>n=4550	209.7±1.95<br>n=1215	226.7±2.09<br>n=1065	192.2±2.23<br>n=961	210.8±2.38<br>n=1061
	p<0.0001 (MW)		p<0.0001 (MW)		p<0.0001 (MW)	

Supplementary Figure S1

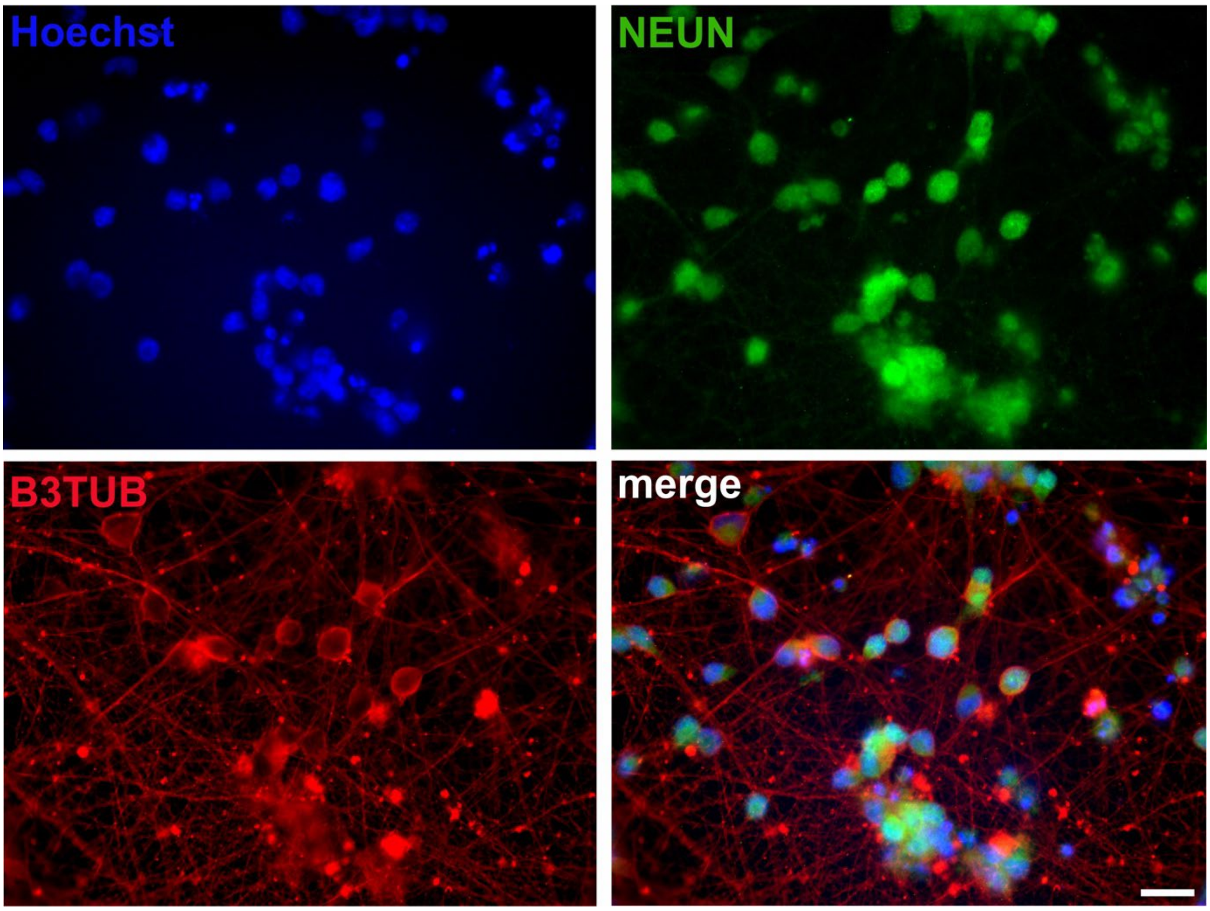

Supplementary Figure S1: Percentage of NeuN positive cells in control neurons (DIV21) stained with neuronal specific beta 3 tubulin antibody. Nuclei were stained using Hoechst. Scale bar 20 µm.

104 cells were counted from 3 separate immunostainings. 93 cells were positive for NeuN (corresponding to 89%).

Supplementary Figure S2

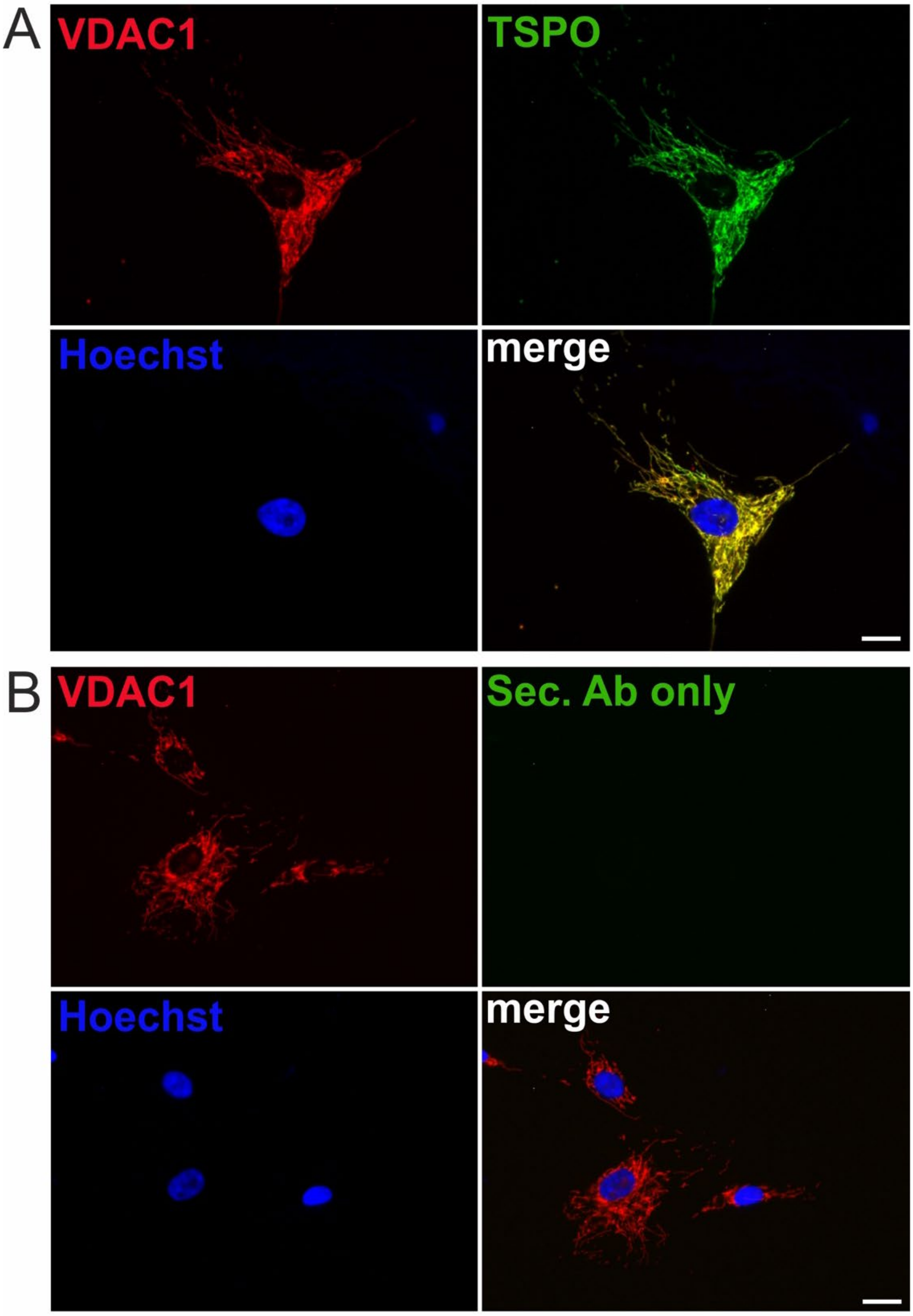

Supplementary Figure S2: Control for TSPO antibody specificity. (A) control astrocytes were stained with antibodies against VDAC1 and TSPO. (B) Control astrocytes were stained using anti VDAC1 antibody, and TSPO antibody was omitted. Nuclei were stained using Hoechst. Scale bar 20 μm.
